# Influence of Fruit and Vegetable Consumption on Antioxidant Status and Semen Quality: A Cross-Sectional Study in Adult Men

**DOI:** 10.3389/fnut.2021.753843

**Published:** 2021-10-15

**Authors:** Dawid Madej, Dominika Granda, Ewa Sicinska, Joanna Kaluza

**Affiliations:** Department of Human Nutrition, Institute of Human Nutrition Sciences, Warsaw University of Life Sciences–SGGW, Warsaw, Poland

**Keywords:** antioxidant defenses, blood, semen, infertility, fruits, vegetables

## Abstract

The influence of fruit and vegetable consumption on semen quality by reducing oxidative stress is inconsistent. Thus, the association between the consumption of these products, antioxidant status, and semen quality was investigated in 90 men aged 18–40. The consumption of fruit and vegetables was collected using the 3-day food record method. Antioxidant status: total antioxidant capacity in semen (TAC-s) and blood (TAC-b), blood superoxide dismutase (SOD-b), glutathione reductase (GR-b), glutathione peroxidase (GPx-b), catalase (CAT-b) activity, and malondialdehyde concentration in blood (MDA-b) were measured. Sperm concentration, leukocytes in the ejaculate, vitality, motility, and sperm morphology were examined using computer-aided semen analysis (CASA). The consumption of fruit and vegetables was positively correlated with sperm concentration, vitality, motility, TAC-s, TAC-b, and SOD-b activity. The TAC-s and TAC-b were positively related to motility, TAC-s was inversely correlated with sperm tail defects. The SOD-b activity was positively correlated with vitality, motility, sperm morphology, and inversely with sperm tail defects and leukocytes in the ejaculate. Compared to the men in the first quartile of fruit and vegetable consumption (<318 g/day), those in the highest quartile (>734 g/day) had the highest sperm concentration, vitality, motility, TAC-s, TAC-b, GPx-b activity, and the lowest MDA-b concentration (based on multivariate regression models). A high consumption of fruit and vegetables may positively influence selected sperm quality parameters by improving the antioxidant status of semen and blood.

## Introduction

Results of epidemiological studies confirm the growing problem of idiopathic infertility in men. It is estimated that the problem of male infertility may affect up to 50% of couples in the world; however, this value may be underestimated because it does not reflect all world regions (1). Many factors contribute to its development, i.a. environmental pollution, lifestyle, chronic diseases, and oxidative stress (2–4).

Reactive oxygen species (ROS) play a physiological role in spermatogenesis, i.a. they regulate the sperm maturation process and ability to fertilize (5–7). However, their excess can cause oxidative stress. Long-term oxidative stress leads to reproductive cell damage, including increased sperm cell membrane lipid peroxidation, decreased motility, and damaged sperm DNA. Moreover, it may increase the number of leukocytes in semen and lead to hormone fluctuations (e.g., testosterone), causing disorders in the functioning of the male reproductive system and significantly reducing the ability to fertilize (3, 8–10). Thus, assessing the level of oxidative stress in the body should be included in male infertility diagnosis, and may be a useful tool in determining the ability to fertilize sperm (11).

The defense system, against ROS in male reproductive cells includes antioxidant enzymes and non-enzymatic substances like vitamin C, A, β-carotene, folate, and polyphenols, present among others in fruit and vegetables (6, 12, 13). Many studies confirm the beneficial effect of specific antioxidant nutrients on improving the male reproductive system's antioxidant status, sperm quality, and support in treating idiopathic infertility (14–19). Also, the dietary fiber present in fruit and vegetables may reduce oxidative stress by participating in the assimilation of polyphenols and carotenoids in the intestine and modulating the immune system's response by having a positive impact on the gut microbiome (20, 21). Some studies indicated that the consumption of fruit and vegetables, as well as pro-healthy eating patterns (frequent consumption of i.a. fruit, vegetables, whole-grain products, legumes, and nuts), may be connected with better semen quality (22–27). In contrast, in other studies, a beneficial influence of pro-healthy dietary patterns on the quality of semen was not observed (28, 29), and results on associations between dietary fiber intake and hormone levels affecting the male reproductive system were inconsistent (30–34). These inconsistent results may be down to the effect of the presence of pesticide residues, heavy metals and nitrate in fruit and vegetables, substances that can cause the generation of ROS, and have a proven negative impact on sperm quality (3, 35–37).

Considering that the influence of fruit and vegetable consumption on semen quality is inconsistent and no study has analyzed their consumption in relation to antioxidant status and semen quality simultaneously, this study aimed to examine the association between fruit and vegetable consumption, antioxidant status, and semen quality in adult men.

## Materials and Methods

### Participants and Study Design

The cross-sectional study was conducted on 90 men, aged 18–40 years old, from 2018 to 2019. Men were recruited based on voluntary applications. Information encouraging participation was disseminated on social media, in gyms, and in sports clubs located in Warsaw. Inclusion criteria were: male gender and age (18–40). The criteria of exclusion were: diagnosed infertility or sterility, permanent injuries, acute or chronic reproductive system diseases, surgery of reproductive organs within the past year, diagnosed hormonal disorders or hormone therapy within the last year, chemotherapy, radiotherapy, a disease requiring special dietary treatment, or limitation of legal capacity. All participants gave written informed consent to participate in the study prior to inclusion. The study was approved by the Ethical Committee at the Institute of Human Nutrition Sciences, Warsaw University of Life Sciences (N08/2016) and was performed in accordance with ethical standards laid down in the 1964 Declaration of Helsinki and its later amendments.

A self-administered questionnaire was used to collect information on sociodemographic and lifestyle factors like sleeping time, smoking cigarettes, alcohol consumption, and using supplements. Weight and height were measured with a calibrated scale and stadiometer with an accuracy of 0.1 kg and 0.1 cm, respectively. The data was used to calculate the Body Mass Index (BMI) as weight/height^2^ (kg/m^2^). Physical activity (MET-h/week) was assessed base on the long version of the validated International Physical Activity Questionnaire (IPAQ), which considers the average time and frequency of individual activities related to everyday life and sports (38).

### Fruit and Vegetable Consumption

Fruit and vegetable consumption as well as energy intake were assessed using the 3-day food record method covering two weekdays and one weekend day. During the same week as the diet data, blood collection, and sperm donation were conducted. Before filling in the food records, the participants were trained how to record detailed information about the foods and drinks consumed, preparation meals methods and recipes, and how to determine the estimate portion size by using kitchen scales and household measures (i.e., spoons, cups, bowls), and were given an album of photographs of food products and dishes. A qualified interviewer accurately checked the returned questionnaires, and possible missing or imprecise information about the food consumed and portion sizes were completed. The obtained data were entered into the “Dieta 6” software developed by the National Institute of Public Health–National Institute of Hygiene in Poland (NIPH–NIH). The software contains current food composition tables also developed by NIPH–NIH.

### Collection and Blood Analysis

The fasting blood samples were collected in the morning by a registered nurse at a clinic. Anticoagulant (heparin or EDTA) tubes were used to obtain whole blood samples. The tubes without anticoagulant were centrifuged at 2,000 × g for 15 min and at 4°C to obtain the serum. All blood samples were partitioned and stored at −80°C for further analysis. The hemoglobin level in whole blood, as well as the concentration of total testosterone, prolactin, the follicle-stimulating hormone (FSH), and high-sensitivity C-reactive protein (hs-CRP) in serum, were determined by a certified laboratory. The assay kits (Roche, Switzerland) and Cobas 8000 analyser (Roche, Switzerland) were used to measure the hormone level using the electrochemiluminescence method, and the hs-CRP by applying the immunoturbidimetric method.

### Semen Analysis

**Figure 1:**
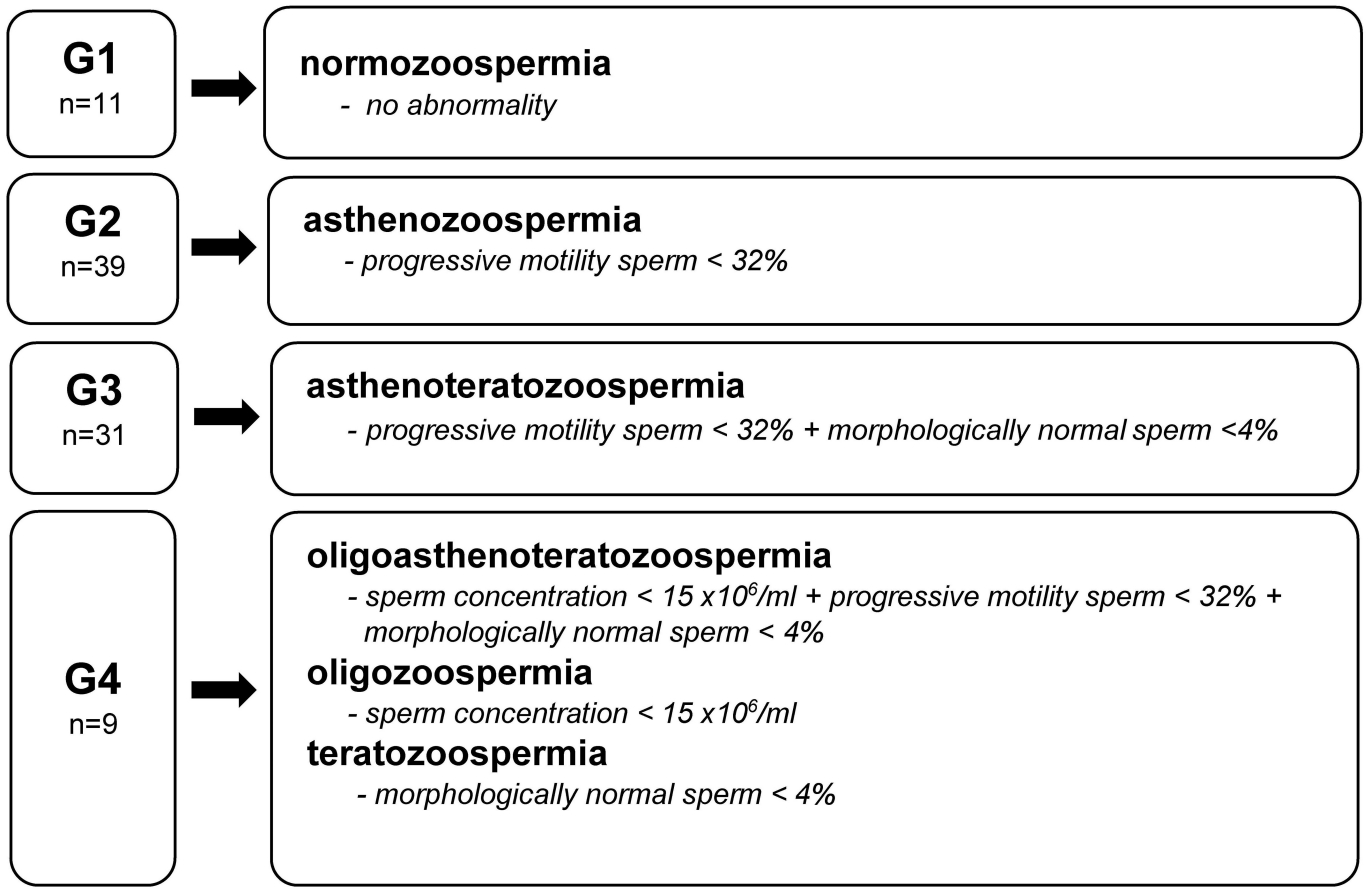
Groups (G) of men by semen diagnosis and WHO reference value (39).

The semen samples were obtained by masturbation into a sterile container in a certified laboratory's private room 2–7 days after last ejaculation. Analyses were performed according to the World Health Organization (WHO) laboratory manual for the examination and processing of human semen by an experienced technician (39). The sperm concentration, amount of leukocytes in the ejaculate, vitality, total and progressive motility, sperm morphology, and sperm head and tail defects were examined using computer-aided semen analysis (CASA) (SCA Scope, Microptic, Spain). The obtained results were compared to the reference values presented by the WHO (39). Based on the diagnosis of an embryologist and WHO semen quality nomenclature (39), the studied men were divided into four groups (Figure 1). Group 1 (G1) included men with no abnormalities in sperm quality. Group 2 (G2) included those with demonstrated asthenozoospermia, while Group 3 (G3) were men with asthenoteratozoospermia. The remaining men with diagnosed oligoasthenoteratozoospermia, oligozoospermia, and teratozoospermia were included in Group 4 (G4).

Parallel to quality analysis, the total antioxidant capacity in semen (TAC-s) was assessed (the methodology was presented in the section Antioxidant Status).

### Antioxidant Status

The measurement of total antioxidant capacity in semen (TAC-s) and blood serum (TAC-b) was based on the Trolox equivalent antioxidant capacity method (TEAC) and was determined using an assay kit (Randox Laboratories Ltd., UK). This colorimetric method allows to determine the capacity of a sample's antioxidants to inhibit ABTS+∙ (radical monocation of 2,20-azinobis-3-ethylbenzothiazoline-6-sulphonic acid). The results were compared to the Trolox (soluble in water analog vitamin E), which constitutes a reference antioxidant standard.

Ready-to-use kits (Randox Laboratories Ltd., UK) were used to determine superoxide dismutase (SOD-b) and glutathione reductase (GR-b) activity in the blood. For this purpose, the samples of EDTA whole blood were centrifuged (10 min at 3,000 rpm) to separate plasma from erythrocytes. The received erythrocytes were washed four times with 0.9% NaCl, diluted in 0.01 mol/l phosphate buffer and incubated (37°C) with xanthine oxidase and INT (2-(4-indophenyl)-5-phenyltetrasodium chloride) to determine SOD-b activity, and with NADPH (nicotinamide adenine dinucleotide phosphate) to determine GR-b activity. Enzyme activity was determined spectrophotometrically (505 and 340 nm, respectively) based on the degree of inhibition of this reaction (Multiskan Go, Thermo Scientific, USA).

Glutathione peroxidase (GPx-b) activity in the whole blood was determined using a RANSEL kit (Randox Laboratories Ltd., UK). The samples, diluted in Drabkin's reagent, were incubated at 37°C with cumene hydroperoxide. The decrease of absorbance over time was measured at 340 nm (MultiskanGo, Thermo Scientific, USA).

The level of decomposition of hydrogen peroxide (Sigma-Aldrich, US) was a measure of catalase activity in blood serum (CAT-b). The intensity of the colored complex formed by the mixture of purpald (4-amino-3-hydrazino-5-mercapto-1,2,4-triazole measured) and formaldehyde (Sigma-Aldrich, US) was measured using the spectrophotometric method (wavelength of 340 nm, MultiskanGo, Thermo Scientific, USA) (40, 41).

To determine lipid peroxidation levels, the concentration of malondialdehyde (MDA) in blood serum was measured using the spectrophotometric method (wavelength of 530 nm, MultiskanGo, Thermo Scientific, USA). This naturally occurring product of lipid peroxidation in reaction with thiobarbituric acid (TBA) at high temperatures (90–100°C) formed a color complex—thiobarbituric acid-reactive substances (TBARS) (42, 43).

### Statistical Analysis

Statistical analyses were performed using Statistica software (version 13.4, StatSoft, USA). The characteristics data were shown as mean values ± standard deviation (SD) and as percentages of men by smoking status, alcohol consumption and supplements usage. The results were presented separately for four groups of men, which were created based on their semen quality (Figure 1).

The Chi-square test was used to determine statistically significant differences between categorical variables and groups of men with different semen quality. Based on the Shapiro-Wilks test, the hypothesis of normality of continuous variables was rejected; therefore, the Kruskal-Wallis and Mann-Whitney U-tests were used to determine statistically significant differences between groups.

After transforming data [log(*x*)] to obtain normal distributions, Pearson correlation coefficients were used to determine associations between fruit and vegetable consumption and antioxidant status vs. the parameters of semen quality. Partial correlations were assessed to take into account the potential effect of the following parameters on the observed associations: age (years, continuous), BMI (<18.5, 18.5–24.9 and >24.9 kg/m^2^), sitting time (h/d, continuous), physical activity level (MET-h/week, continuous), smoking (ever, never), alcohol consumption (ever, never), supplement use (yes, no), wearing briefs and boxer shorts (ever, never), and energy intake (kcal/day, continuous).

Based on multivariate regression models adjusted for the same variables as partial correlations, antioxidant status and semen quality parameters were presented by quartiles of fruit and vegetable consumption (mean ± standard error). The results with *P*-values of ≤ 0.05 were admitted as statistically significant.

## Results

### Characteristics of the Studied Population

The characteristics of the studied men by semen quality were presented in Table 1. Only 12% of men had proper sperm quality. Asthenozoospermia was diagnosed in 43% of men, asthenoteratozoospermia in 35%, while oligoasthenoteratozoospermia, oligozoospermia, or teratozoospermia were observed in the remaining 10%. A statistically significantly higher consumption of fruit and vegetables was observed in men without abnormalities in sperm quality (G1) compared to the other groups of men (G2, G3, and G4). When fruit and vegetables were investigated separately, similar results were found for both fruit and vegetable consumption.

**Table 1:** Baseline characteristics of participants (mean ± SD).

**Variables**	**G1 (***n*** = 11)**	**G2 (***n*** = 39)**	**G3 (***n*** = 31)**	**G4 (***n*** = 9)**	***P*** **-value**
Consumption (g/day)					
Fruit and vegetables	874 ± 321^a^	496 ± 308^b^	554 ± 290^b^	541 ± 224^b^	***0.007*** *
Fruit	445 ± 258^a^	176 ± 173^b^	193 ± 191^b^	259 ± 200^b^	***0.016*** *
Vegetables	430 ± 153^a^	320 ± 201^b^	361 ± 159^ab^	281 ± 127^b^	***0.050*** *
Age (years)	25.4 ± 4.1	28.3 ± 5.6	27.5 ± 4.6	27.1 ± 3.2	0.475*
BMI (kg/m^2^)	23.7 ± 2.7	25.4 ± 5.3	24.8 ± 3.7	25.1± 3.5	0.661*
Sleeping time (h/day)	7.1 ± 0.5	7.2 ± 0.8	7.2 ± 0.8	7.1 ± 1.0	0.944*
Sitting time (h/day)	9.3 ± 2.2	7.9 ± 2.5	8.0 ± 2.3	8.4 ± 1.8	0.301*
Physical activity (MET-h/week)	112 ± 52	140 ± 81	146 ± 66	105 ± 24	0.221*
Current smokers (%)	18	18	23	0	*0.492^#^*
Alcohol consumption (%)
>2–3 times/week	91	87	87	100	*0.712^#^*
≤ 1 times/week or never	9	13	13	0	
Supplement use (%)	64	72	65	44	*0.484^#^*
Type of underwear (%)					
Briefs and boxer shorts	91^a^	71^b^	97^a^	89^a^	***0.032*** **^#^**
Shorts	9	29	3	11	
Energy intake (kcal/day)	2,527 ± 703	2,417 ± 586	2,583 ± 823	2,340 ± 492	*0.841*
Blood analysis					
Hemoglobin (g/dl)	15.6 ± 0.8	15.5 ± 0.8	15.3 ± 1.0	15.4 ± 0.7	0.687*
Total testosterone (ng/dl)	564 ± 118	517 ± 147	529 ± 201	435 ± 195	0.243*
FSH (mlU/ml)	4.9 ± 1.8	5.6 ± 2.0	5.0 ± 2.3	5.3 ± 1.7	0.602*
Prolactin (ng/dl)	9.6 ± 1.4	9.1 ± 2.8	8.8 ± 3.2	9.9 ± 2.3	0.555*
hCRP (mg/dl)	1.3 ± 1.0	1.5 ± 1.9	0.8 ± 0.8	0.7 ± 0.3	0.426*

*G1, normozoospermia; G2, asthenozoospermia; G3, asthenoteratozoospermia; G4, oligoasthenoteratozoospermia, oligozoospermia, and teratozoospermia; FSH, follicle-stimulating hormone; hCRP, high-sensitivity C-reactive protein; MET, metabolic equivalent of task*;

* *Kruskal-Wallis test*;

# *Chi^2^ test*;

a, b * different letters indicate statistically significant differences between a group, p-value ≤ 0.05 (Mann–Whitney U-test). The results with P-values of ≤ 0.05 were admitted as statistically significant and were marked in bold cursive*.

Significantly fewer men in the G2 group declared wearing briefs and boxer shorts than men in other groups. Men classified into different semen quality groups did not differ in terms of age, BMI, sleeping time, sitting time, physical activity, smoking status, alcohol consumption, supplement use, and energy intake. Moreover, no statistically significant differences in hemoglobin levels, analyzed hormones, and hsCRP were observed between the groups of men.

### Semen Quality and Antioxidant Status

Semen analysis showed the highest average sperm concentration in the G1 group and a statistically significantly higher percentage of morphologically normal sperm in the G1 and G2 groups compared to other groups (Table 2). In the G1 and G2 groups, a significantly lower percentage of sperm head defects vs. the G3 and G4 groups were observed (88 and 91% vs. 98 and 97%, respectively). A significantly higher percentage of motile spermatozoa (total and progressive motility) was observed in the G1 and G4 groups than in the G2 and G3 groups. However, the groups of men did not differ statistically significantly in terms of sperm viability, the number of leucocytes in the ejaculate, and the percentage of sperm tail defects.

**Table 2:** Semen quality parameters (mean ± SD).

**Variables**	**G1 (***n*** = 11)**	**G2 (***n*** = 39)**	**G3 (***n*** = 31)**	**G4 (***n*** = 9)**	***P*** -value*
Sperm concentration (× 10^6^/ml)	93.1 ± 53.0^a^	82.9 ± 61.4^ab^	76.8 ± 49.9^b^	33.2 ± 48.4^b^	***0.030***
Leukocytes in ejaculate (× 10^6^/ml)	0.2 ± 0.1	0.5 ± 0.4	0.5 ± 0.3	0.3 ± 0.4	*0.183*
Vitality (live spermatozoa, %)	79.4 ± 6.9	71.7 ± 9.9	74.1 ± 9.1	69.6 ± 7.2	*0.087*
Total motility (PR + NP, %)	55.5 ± 7.3^a^	28.1 ± 10.0^b^	27.5 ± 11.0^b^	48.2 ± 16.3^a^	*** <0.001***
Progressive motility (%)	39.7 ± 9.1^a^	12.9 ± 7.6^b^	10.9 ± 7.0^b^	31.2 ± 13.2^a^	*** <0.001***
Sperm morphology (normal forms, %)	10.4 ± 6.0^a^	7.9 ± 3.1^a^	1.9 ± 1.3^b^	3.4 ± 4.9^b^	*** <0.001***
Sperm head defects (%)	88.3 ± 5.5^a^	90.6 ± 3.7^a^	97.7 ± 1.7^b^	96.6 ± 4.9^b^	*** <0.001***
Sperm tail defects (%)	5.9 ± 3.7	7.0 ± 5.8	8.6 ± 7.1	8.4 ± 7.8	*0.730*

*G1, normozoospermia; G2, asthenozoospermia; G3, asthenoteratozoospermia; G4, oligoasthenoteratozoospermia, oligozoospermia, and teratozoospermia*;

* *Kruskal-Wallis test*;

a, b * different letters indicate statistically significant differences between a group; p-value ≤ 0.05 (Mann–Whitney U-test). The results with P-values of ≤ 0.05 were admitted as statistically significant and were marked in bold cursive*.

Moreover, in comparison to the groups of men with sperm abnormalities (G2, G3, and G4), the group of men with normozoospermia (G1) had the statistically significantly highest TAC levels in semen and blood as well as SOD-b activity (Table 3). The groups of men did not differ in terms of GPx-b, GR-b, CAT-b activities, and MDA-b concentration.

**Table 3:** Antioxidant status parameters (mean ± SD).

**Variables**	**G1 (***n*** = 11)**	**G2 (***n*** = 39)**	**G3 (***n*** = 31)**	**G4 (***n*** = 9)**	***P*** -value*
TAC-s (mmol/l)	1.4 ± 0.2^a^	1.2 ± 0.2^b^	1.2 ± 0.1^b^	1.1 ± 0.2^b^	***0.003***
TAC-b (mmol/l)	1.9 ± 0.2^a^	1.7 ± 0.2^b^	1.7 ± 0.2^b^	1.7 ±0.1^b^	***0.007***
SOD-b (U/g Hb)	1,827 ± 88^a^	1,317 ± 182^b^	1,338 ± 201^b^	1,353 ± 177^b^	*** <0.001***
GPx-b (U/g Hb)	56.4 ± 9.8	49.6 ± 11.6	49.6 ± 10.9	51.9 ± 10.7	*0.224*
GR-b (U/g Hb)	13.0 ± 4.2	12.9 ± 3.9	12.8 ± 3.3	11.7 ± 2.6	*0.831*
CAT-b (nmol/min/ml)	14.2 ± 2.1	12.5 ± 3.9	13.1 ± 2.6	11.8 ± 2.7	*0.418*
MDA-b (μmol/ml)	2.5 ± 0.4	2.7 ± 0.4	2.6 ± 0.3	2.8 ± 0.3	*0.102*

*G1, normozoospermia; G2, asthenozoospermia; G3, asthenoteratozoospermia; G4, oligoasthenoteratozoospermia, oligozoospermia, and teratozoospermia; TAC-s, total antioxidant capacity in semen; TAC-b, total antioxidant capacity in blood; SOD-b, superoxide dismutase activity in blood; GPx-b, glutathione peroxidase activity in blood; GR-b, glutathione reductase activity in blood; CAT-b, catalase activity in blood; MDA-b, malondialdehyde concentration in blood*;

* *Kruskal-Wallis test*;

a, b * different letters indicate statistically significant differences between a group; p-value ≤ 0.05 (Mann–Whitney U-test). The results with P-values of ≤ 0.05 were admitted as statistically significant and were marked in bold cursive*.

### Fruit and Vegetable Consumption in Relation to Antioxidant Status and Semen Quality Parameters

The results of the Pearson correlation between fruit and vegetable consumption, antioxidant status, and selected semen quality parameters were shown in Table 4. The consumption of fruit and vegetables was statistically significantly positively correlated with sperm concentration (*r* = 0.30), vitality (*r* = 0.28), as well as total and progressive motility (*r* = 0.38 and 0.43, respectively). Significant positive correlations were also found between the consumption of fruit and vegetables vs. TAC-s (*r* = 0.42), TAC-b (*r* = 0.28), and SOD-b activity (*r* = 0.29).

**Table 4:** Pearson correlation coefficients between fruit and vegetable consumption, antioxidant status and selected semen quality parameters (r, *p*).

**Variables**	**Fruit and vegetable consumption (g/day)**		**TAC-s (mmol/l)**		**TAC-b (mmol/l)**		**SOD-b (U/g Hb)**		**GPx-b (U/g Hb)**		**GR-b (U/g Hb)**		**CAT-b (nmol/min/ml)**		**MDA-b (μmol/ml)**	
	**Crude**	Partial*	**Crude**	Partial*	**Crude**	Partial*	**Crude**	Partial*	**Crude**	Partial*	**Crude**	Partial*	**Crude**	Partial*	**Crude**	Partial*
Fruit and vegetable consumption (g/day)	–	–	**0.36** ***p*** **< 0.001**	**0.42** ***p*** **< 0.001**	**0.25** ***p*** **= 0.02**	**0.28** ***p*** **= 0.01**	**0.27** ***p*** **= 0.01**	**0.29** ***p*** **= 0.01**	0.13 *p* = 0.24	0.12 *p* = 0.29	0.05 *p* = 0.65	0.02 *p* = 0.86	0.14 *p* = 0.18	0.17 *p* = 0.14	−0.17 *p* = 0.13	−0.16 *p* = 0.18
Sperm concentration(× 10^6^/ml)	**0.28** ***p*** **= 0.01**	**0.30** ***p*** **= 0.02**	0.00 *p* = 0.99	0.01 *p* = 0.96	−0.04 *p* = 0.75	−0.04 *p* = 0.75	0.08 *p* = 0.44	0.11 *p* = 0.35	0.05 *p* = 0.65	0.44 *p* = 0.60	0.08 *p* = 0.48	0.03 *p* = 0.79	0.03 *p* = 0.76	0.09 *p* = 0.45	−0.04 *p* = 0.74	−0.04 *p* = 0.74
Leukocytes in ejaculate (× 10^6^/ml)	−0.07 *p* = 0.49	−0.05 *p* = 0.67	0.13 *p* = 0.22	0.13 *p* = 0.27	−0.09 *p* = 0.44	−0.11 *p* = 0.36	**−0.22** ***p*** **= 0.04**	**−0.27** ***p*** **= 0.02**	−0.09 *p* = 0.37	−0.18 *p* = 0.12	0.06 *p* = 0.59	0.08 *p* = 0.49	−0.08 *p* = 0.46	−0.11 *p* = 0.36	0.02 *p* = 0.83	0.04 *p* = 0.72
Vitality(live spermatozoa, %)	**0.25** ***p*** **= 0.02**	**0.28** ***p*** **= 0.01**	0.17 *p* = 0.11	0.17 *p* = 0.14	0.11 *p* = 0.30	0.06 *p* = 0.61	**0.28** ***p*** **= 0.007**	**0.31** ***p*** **= 0.007**	−0.09 *p* = 0.40	−0.11 *p* = 0.35	0.11 *p* = 0.33	0.16 *p* = 0.18	0.02 *p* = 0.86	−0.02 *p* = 0.90	−0.04 *p* = 0.74	−0.18 *p* = 0.12
Total motility (PR + NP, %)	**0.35** ***p*** **< 0.001**	**0.38** ***p*** **= 0.001**	**0.36** ***p*** **= 0.001**	**0.32** ***p*** **= 0.005**	**0.21** ***p*** **= 0.04**	**0.22** ***p*** **= 0.05**	**0.51** ***p*** **< 0.001**	**0.46** ***p*** **< 0.001**	0.03 *p* = 0.78	0.02 *p* = 0.88	−0.005 *p* = 0.96	0.03 *p* = 0.81	0.09 *p* = 0.41	0.09 *p* = 0.44	−0.19 *p* = 0.09	−0.20 *p* = 0.08
Progressive motility(%)	**0.41** ***p*** **< 0.001**	**0.43** ***p*** **< 0.001**	**0.37** ***p*** **< 0.001**	**0.35** ***p*** **= 0.002**	**0.25** ***p*** **= 0.02**	**0.27** ***p*** **= 0.02**	**0.57** ***p*** **< 0.001**	**0.49** ***p*** **< 0.001**	−0.004 *p* = 0.97	−0.007 *p* = 0.95	−0.03 *p* = 0.78	−0.08 *p* = 0.50	0.09 *p* = 0.43	0.11 *p* = 0.33	−0.09 *p* = 0.42	−0.09 *p* = 0.43
Sperm morphology (normal forms, %)	0.02 *p* = 0.89	0.02 *p* = 0.83	0.04 *p* = 0.73	0.09 *p* = 0.44	−0.01 *p* = 0.94	−0.04 *p* = 0.76	**0.22** ***p*** **= 0.03**	**0.20** ***p*** **= 0.05**	0.07 *p* = 0.53	0.09 *p* = 0.41	−0.09 *p* = 0.44	−0.15 *p* = 0.19	−0.12 *p* = 0.27	−0.08 *p* = 0.50	−0.01 *p* = 0.93	0.02 *p* = 0.86
Sperm head defects (%)	−0.01 *p* = 0.93	0.01 *p* = 0.96	−0.02 *p* = 0.84	−0.05 *p* = 0.67	−0.02 *p* = 0.85	0.02 *p* = 0.88	−0.20 *p* = 0.06	−0.19 *p* = 0.10	−0.14 *p* = 0.21	−0.16 *p* = 0.17	0.02 *p* = 0.87	0.05 *p* = 0.69	0.10 *p* = 0.36	0.07 *p* = 0.54	−0.04 *p* = 0.32	0.01 *p* = 0.98
Sperm tail defects (%)	−0.09 *p* = 0.39	−0.14 *p* = 0.23	**−0.24** ***p*** **= 0.03**	**−0.25** ***p*** **= 0.03**	−0.07 *p* = 0.54	−0.06 *p* = 0.61	**−0.25** ***p*** **= 0.02**	**−0.27** ***p*** **= 0.02**	0.05 *p* = 0.67	0.05 *p* = 0.65	0.01 *p* = 0.99	−0.06 *p* = 0.58	−0.12 *p* = 0.27	−0.15 *p* = 0.19	−0.06 *p* = 0.84	0.04 *p* = 0.43

* *In contrast to the crude (not adjusted) the partial correlation coefficients were adjusted for age (years, continuous), BMI (<18.5, 18.5–24.9, and >24.9, kg/m^2^), sitting time (h/d, continuous), physical activity level (MET-h/week, continuous), smoking (ever, never), alcohol consumption (ever, never), supplement use (yes, no), wearing briefs and short boxer (ever, never), and energy (kcal/d, continuous); TAC-s, total antioxidant capacity in semen; TAC-b, total antioxidant capacity in blood; TAC-s, total antioxidant capacity in semen; TAC-b, total antioxidant capacity in blood; SOD-b, superoxide dismutase activity in blood; GPx-b, glutathione peroxidase activity in blood; GR-b, glutathione reductase activity in blood; CAT-b, catalase activity in blood; MDA-b, malondialdehyde concentration in blood; PR, progressive motility; NP, non-progressive motility. The results with P-values of ≤ 0.05 were admitted as statistically significant and were marked in bold cursive*.

Moreover, the TAC-s and TAC-b were positively related to total and progressive motility (partial *r* ranged from 0.22 to 0.35). The TAC-s was inversely correlated with sperm tail defects (*r* = −0.25). A positive correlation was found between SOD-b activity vs. vitality (*r* = 0.31), total and progressive motility (*r* = 0.46 and 0.49, respectively) and morphologically normal sperm (*r* = 0.20). An inverse correlation of sperm tail defects (*r* = −0.27) and leukocytes in ejaculate (*r* = −0.27) with SOD-b activity were observed.

**Figure 2:**
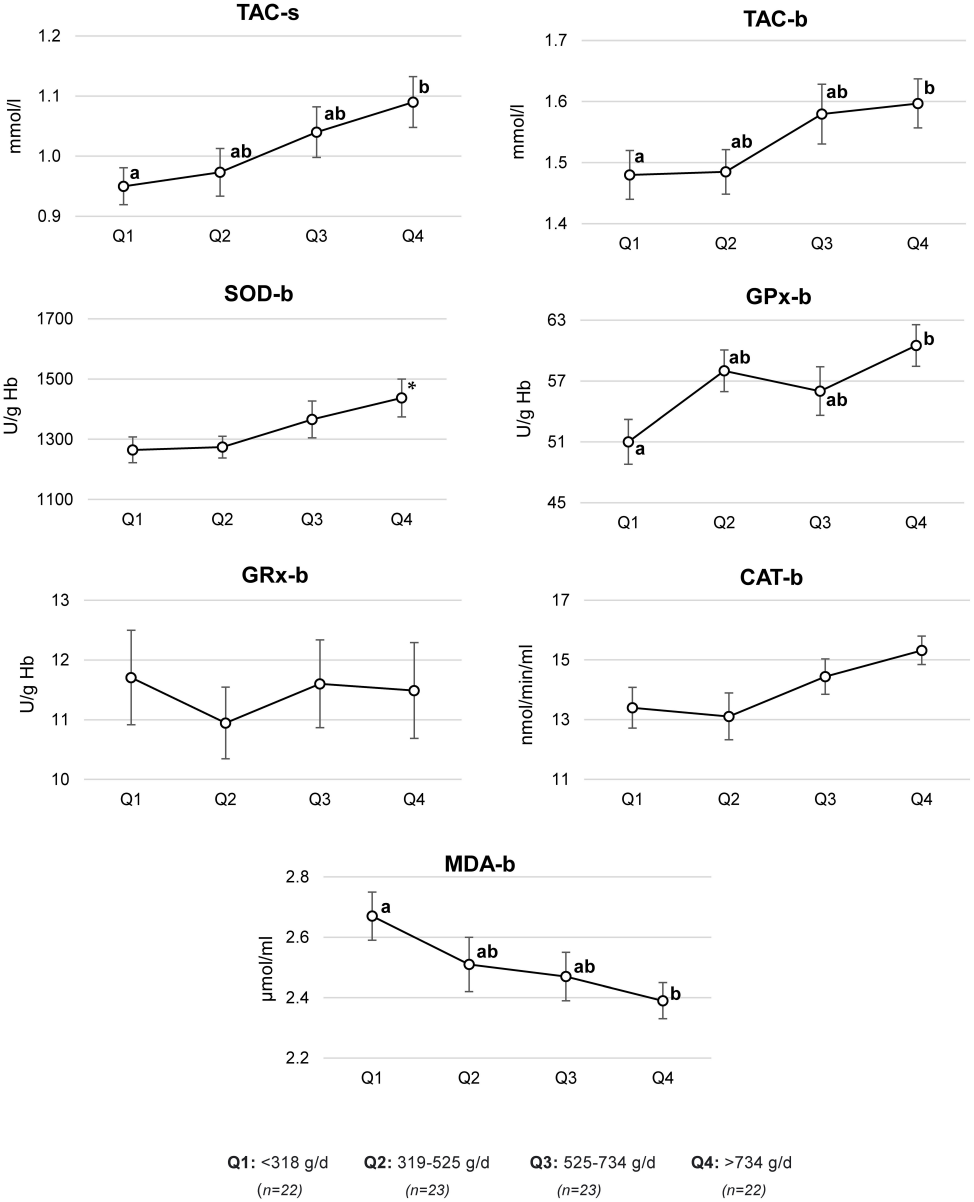
Antioxidant status parameters by quartiles (Q) of fruit and vegetable consumption. Mean ± SE of antioxidant status parameters were estimated based on a multivariate regression model adjusted for age (years, continuous), BMI (<18.5, 18.5–24.9, and >24.9 kg/m^2^), sitting time (h/d, continuous), physical activity level (MET-h/week, continuous), smoking (ever, never), alcohol consumption (ever, never), supplement use (yes, no), wearing briefs and boxer shorts (ever, never), and energy (kcal/d, continuous); TAC-s, total antioxidant capacity in semen; TAC-b, total antioxidant capacity in blood; SOD-b, superoxide dismutase activity in blood; GPx-b, glutathione peroxidase activity in blood; GR-b, glutathione reductase activity in blood; CAT-b, catalase activity in blood; MDA-b, malondialdehyde concentration in blood; ^*a,b*^ different letters indicate statistically significant differences between quartiles; *p*-value ≤ 0.05 (Mann–Whitney U-test); *p-trend between Q1 and Q4, *p*-value = 0.08 (Mann–Whitney U-test).

Based on multivariate regression models, adjusted means of antioxidant status parameters across the quartiles of fruit and vegetable consumption were estimated (Figure 2). Compared to the men in the lowest quartile of fruit and vegetable consumption (<318 g/day), the men in the highest quartile (>734 g/day) had a higher mean of TAC-s (mean ± SE, 0.95 ± 0.03 vs. 1.09 ± 0.04 mmol/l), TAC-b (1.48 ± 0.04 vs. 1.60 ± 0.04 mmol/l), GPx-b (51.0 ± 2.2 vs. 60.5 ± 2.1 U/g Hb), and a lower MDA-b (2.40 ± 0.06 vs. 2.67 ± 0.08 μmol/ml). A trend to a higher mean of SOD-b in men with the highest compared to those with the lowest fruit and vegetable intake was found (1,437 ± 63 vs. 1,265 ± 43 U/g Hb).

**Figure 3:**
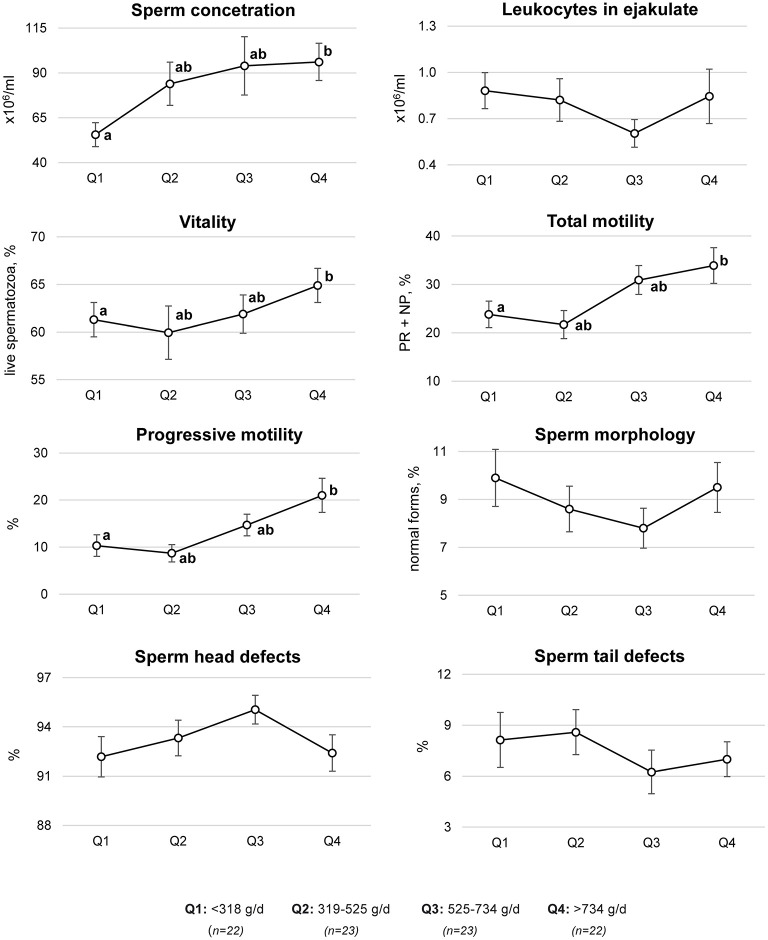
Semen quality parameters by quartiles (Q) of fruit and vegetable consumption. Mean ± SE of semen quality parameters were estimated based on a multivariate regression model adjusted for age (years, continuous), BMI (<18.5, 18.5–24.9, and >24.9 kg/m^2^), sitting time (h/d, continuous), physical activity level (MET-h/week, continuous), smoking (ever, never), alcohol consumption (ever, never), supplement use (yes, no), wearing briefs and boxer shorts (ever, never), and energy (kcal/d, continuous). PR, progressive motility; NP, non-progressive motility; ^*a,b*^ different letters indicate statistically significant differences between quartiles, *p*-value ≤ 0.05 (Mann–Whitney U-test).

Using multivariate regression models, significant differences in selected semen quality parameters across the quartiles of fruit and vegetable consumption were shown (Figure 3). Compared to the men in the first quartile of fruit and vegetable consumption (<318 g/day), the men in the highest quartile (>734 g/day) had a statistically significant higher sperm concentration (55.6 ± 6.7 vs. 96.2 ± 10.4 × 106/ml), vitality (61.3 ± 1.8 vs. 64.9 ± 1.8% of live sperm), total motility (23.8 ± 2.7 vs. 33.9 ± 3.7%), and progressive motility (10.3 ± 2.3 vs. 21.0 ± 3.6%).

## Discussion

To the best of our knowledge, this is the first study that has analyzed the associations of fruit and vegetable consumption with antioxidant status and semen quality and confirms relationships between these parameters.

In this study, a significantly higher consumption of fruit and vegetables in men with normal semen quality than men with abnormality cases was shown. Greater consumption of these products was related to higher sperm concentration, vitality as well as total and progressive semen motility. It should be emphasized that the relationships demonstrated in this study were observed between the lowest vs. the highest quartiles of fruit and vegetable consumption, i.e., <318 vs. >734 g/day. As WHO recommends an intake of a minimum of 400 g/day of these products (44), it can be concluded that a higher consumption of fruit and vegetables may have a beneficial effect on semen quality.

Similar results were also shown in other studies but was, nevertheless, observed with a lower consumption of fruit and vegetables than in this study. A beneficial association of fruit consumption (2.33 servings/day, which corresponds to about 186 g/day if 1 serving = 80 g) on total motility as well as consumption of legumes (3.63 servings/day, which corresponds to about 290 g/day) on sperm morphology was observed (26, 27). Moreover, in a case-control study including 241 men, the total fruit and vegetable consumption in the highest vs. lowest tertile (>522 vs. <225 g/day) was related to a lower risk of asthenozoospermia (27). In other studies, a higher sperm concentration, total and progressive sperm motility, testosterone levels, and a lower DNA fragmentation index were associated with a pro-healthy dietary pattern (diet based on frequent consumption of i.a. fruit, vegetables, whole-grain products, legumes, and nuts) (22–25). Additionally, the intake of antioxidants common in fruit and vegetables (such as vitamin C and β-carotene) was related to a high total sperm count, motility, and low damage and fragmentation of sperm DNA, while a low intake of vitamin C, lycopene and folate had a negative association with semen concentration and total motility (45). Moreover, based on intervention studies, it was concluded that vitamins C and E as well as folic acid may improve sperm concentration, motility, and morphology in infertile men (18, 19, 46).

It is well-known that a balanced diet rich in antioxidant substances also plays an important role in the prooxidative-antioxidant balance of the body. The TAC level and antioxidant enzymes activity are significantly related to consumed food ingredients with antioxidant properties such as vitamin C, E, carotenes, polyphenols, and folate. In this study, the TAC in semen and blood and SOD activity in blood were positively associated with fruit and vegetable consumption. Moreover, the semen and blood TAC and GPx blood activity were highest in men characterized by the highest consumption of these products. Also, the lowest concentration of MDA in blood was observed in the same men. The results of intervention studies indicate that a higher consumption of antioxidant nutrients (polyphenols, alpha-lipoic acid, zinc, and folic acid) increased TAC levels in the blood and semen of infertile men (47–49). In another study, antioxidants intake with food and supplements (zinc, vitamin C, vitamin E, and folic acid) improved semen quality (lower level of sperm DNA damage) in 80 men with no reported fertility problem (50). Also, in animal studies, it was found that zinc supplementation increased SOD and GPx activity in the epididymis, liver and intestine and decreased lipid peroxidation levels in the epididymis of rats exposed to oxidative stress caused by high doses of iron (51, 52).

In the present study, higher semen and blood TAC as well as higher blood SOD activity in men with normozoospermia than in men with confirmed abnormalities in semen quality were observed. At the same time, TAC in both semen and blood was positively correlated with total and progressive sperm motility; TAC in semen was inversely correlated with sperm tail defects. Additionally, SOD activity in blood was positively related to vitality, total and progressive motility and morphology, and negatively correlated with the level of leukocytes in the ejaculate. The relationship between TAC level, antioxidant enzymes activity, and fertility were also observed by other authors. Men with astheno- and oligoasthenoteratozoospermia had a lower seminal TAC level and SOD activity compared to normozoospermic men (53–55). In the subfertile men, seminal but not blood TAC was lower than in men with idiopathic infertility (7). Furthermore, in a few studies, semen TAC was positively correlated with sperm concentration, total, and progressive motility as well as sperm morphology (lower percentage of abnormal sperm head and tail) (7, 56, 57). Also, in the animal study, rats exposed to oxidative stress, induced by supplementing high doses of iron, had a lower percentage of live sperm, lower TAC in the prostate, lower SOD, and GPx activities in the epididymis as well as a higher concentration of MDA in the testes (51).

Considering the results of this study and other studies, the conclusion can be drawn that semen TAC can be used as a good biomarker to assess the redox status in semen and identify the defensive ability against oxidative stress of the male reproductive system. The determination of its level should be combined with the measurement of other parameters of semen quality. Moreover, TAC assessment in male infertility may justify nutritional intervention based on increasing antioxidant intake as therapy for some patients (58).

Moreover, fruit and vegetables are one of the main sources of fiber in a diet. It has been shown that dietary fiber may have a beneficial effect on reducing oxidative stress in the body by participating in the assimilation of antioxidants in the intestine present in fruit and vegetables, such as polyphenols and carotenoids (20, 21). Increased dietary fiber consumption also positively impacts the gut microbiome, which plays a key role in the immune system's function and reduces the concentration of pro-inflammatory mediators such as IL-6 and CRP (59, 60). However, the effect of dietary fiber consumption on estrogen and testosterone concentration is not fully understood (30–34).

Although most studies have shown a positive role of fruit and vegetable consumption on male sperm quality, in several of them, no such beneficial associations were observed. Their consumption was not related to any sperm quality indicators (sperm concentration, progressive motility, and sperm morphology) in 188 healthy men aged 18–22 (61). Also, the results of other studies show no associations between a pro-healthy dietary pattern (frequent consumption of i.a. fruit, vegetables, whole-grain products, legumes, and nuts) and semen quality (28, 29). It is worth noting that vegetables and fruit may contain pesticide residue and/or be contaminated with heavy metals. It has been shown that the consumption of fruit and vegetables with high pesticide residue (but not with low or moderate residue) was associated with a lower percentage of morphologically normal sperm and total sperm motility as well as the deterioration of semen antioxidant status (26, 35, 45, 62). Pesticides, including organophosphates, bipyridyl herbicides, and organochlorine, may stimulate an increased production of ROS as well as induce lipid peroxidation and reduce total antioxidant levels in the blood (63). Also, the results of animal and human studies confirm that heavy metals have a negative effect on semen and the male reproductive system (36, 64). Additionally, raw vegetables are the main nitrate source in a usual diet (about 80%) (65). The European Food Safety Authority reported that nitrate, after converting to nitrite (during storage or by oral bacteria and enzymes in salivary), might cause adverse health effects (66). Whereas, in a systematic review including animal studies, a negative effect of the intake of nitrate with water on semen quality parameters was observed (37).

It is also worth noting that some of the investigated semen parameters (sperm morphology and leukocytes in the ejaculate) were not related to the consumption of fruit and vegetables in our study. Independently from antioxidant dietary intake, morphological sperm damage could be generated by other factors like genetic, enzymatic, or ROS-related factors during spermatogenesis (3, 5).

The role and potential mechanisms of some non-dietary lifestyle factors on semen quality are still inconsistent. In this study, we did not observe any differences among groups of men in terms of age, BMI, sleeping time, sitting time, physical activity, smoking status, alcohol consumption, supplement use, and energy intake. Also, in some studies, the impact of sleep duration (67) or physical activity (68, 69) on semen quality was not shown. However, associations between physical activity as well as a sedentary life-style and semen quality in other studies have been found (70, 71).

The strength of the study was the use of the 3-day food record method to collect data about food consumption and assess fruit and vegetable consumption. A qualified interviewer accurately checked the returned questionnaires, and possible missing or imprecise information about the food consumed and portion sizes were completed. Also, this method is not based on the respondents' memory, thus it is free from recall bias. Since data was collected in the early spring and autumn, some restrictions in access to certain fruit and vegetables may have potentially affected their consumption. Another limitation of this study is the number of studied men and that they were not randomly selected but volunteered to participate in the study. However, they were qualified based on strict inclusion and exclusion criteria. Moreover, because of the cross-sectional design of the study, it cannot be predicted if increasing fruit and vegetable consumption has a beneficial effect on antioxidant status and sperm quality but the indicated interrelations might for a basis for intervention in further studies. Also, only one semen sample was analyzed; nonetheless, some authors have shown that one sample is sufficient to assess the quality of semen in epidemiological studies (72).

## Conclusions

In summary, the results indicate that high fruit and vegetable consumption may be positively associated with semen quality, and this relationship seems to be involved in antioxidant status underlying the etiology of sperm quality disorders. Taking into account the results of this study and the results of other authors, it can be concluded that the nutritional strategy based on a high consumption of fruit and vegetables to prevent and treat infertility by improving the redox status should be analyzed in future clinical studies.

## Data Availability Statement

The raw data supporting the conclusions of this article will be made available by the authors, without undue reservation.

## Ethics Statement

The studies involving human participants were reviewed and approved by the Ethical Committee at the Institute of Human Nutrition Sciences, Warsaw University of Life Sciences (N08/2016) and has been performed in accordance with the ethical standards laid down in the 1964 Declaration of Helsinki and its later amendments. The patients/participants provided their written informed consent to participate in this study.

## Author Contributions

DM: conceptualization, methodology, formal analysis, investigation, and writing—original draft. DG, ES and, JK: writing—review and editing and visualization. All authors have read and agreed to the published version of the manuscript.

## Funding

The study was supported by a grant from the National Science Center (NCN), Poland (No. 2018/02/X/NZ9/01103). The research for this study was carried out with the use of research equipment purchased as part of the Food and Nutrition Center—modernization of the WULS campus to create a Food and Nutrition Research and Development Center (CZiZ) co-financed by the European Union from the European Regional Development Fund under the Regional Operational Programme of the Mazowieckie Voivodeship for 2014–2020 (Project No. RPMA.01.01.00-14-8276/17).

## Conflict of Interest

The authors declare that the research was conducted in the absence of any commercial or financial relationships that could be construed as a potential conflict of interest.

## Publisher's Note

All claims expressed in this article are solely those of the authors and do not necessarily represent those of their affiliated organizations, or those of the publisher, the editors and the reviewers. Any product that may be evaluated in this article, or claim that may be made by its manufacturer, is not guaranteed or endorsed by the publisher.
